# To Detect Tainted Milk

**Published:** 1893-11

**Authors:** 


TO DETECT TAINTED MEAT.—The official inspectors in Dresden, Germany, make use of Eber's reagent: a mixture of hydrochloric acid, 1 part; alcohol, 3 parts; and ether, 1 part. With this a glass rod is moistened and held close to the suspected meat. If the polished surface becomes clouded (owing to the formation of ammonia), the meat is declared unfit for food and consigned to the fertilizer factory.—*Druggists' Circular.*

